# Determinants of ectopic pregnancy among pregnant women attending referral hospitals in southwestern part of Oromia regional state, Southwest Ethiopia: a multi-center case control study

**DOI:** 10.1186/s12884-021-03618-7

**Published:** 2021-02-12

**Authors:** Urge Gerema, Tilahun Alemayehu, Getachew Chane, Diliab Desta, Amenu Diriba

**Affiliations:** 1 Department of Biomedical sciences (Anatomy course unit), Jimma University, Jimma, Ethiopia; 2 Department of gynecology and obstetrics, Wellega University, Wellega, Ethiopia

**Keywords:** Ectopic pregnancy, Intrauterine pregnancy, Determinants, Southwest Ethiopia

## Abstract

**Background:**

Ectopic pregnancy is an abnormal condition in which implantation of the blastocyst occurs outside the endometrium of the uterus. It is gynecological important, particularly in the developing world, because of associated with enormous rate of high morbidity, during the first trimester of pregnancy. A better understanding of its risk factors can help to prevent its prevalence. However, the determinants of ectopic pregnancy are not well understood and few researches conducted in our country were based on secondary data covering small scale area. This study aimed to identify determinants of ectopic pregnancy among pregnant women attending referral hospitals in Southwestern part of Oromia regional state, Southwest Ethiopia.

**Methods:**

Hospital-based case control study was employed from June 1 to September 30, 2019. The study was conducted in five referral hospitals in Southwestern part of Oromia regional state. Final sample size includes 59 cases and 118 controls. Data were entered by using Epidata version 3.1 and analyzed using SPSS version 23. Descriptive statistics were used to explore the data. All explanatory variables with *p*-value of < 0.25 in bi-variable analysis, then entered into multivariable logistic regression. Associated factors were identified at 95% confidence interval (*p* < 0.05).

**Results:**

Out of 177 (59 cases and 118 controls) participants, 174 (58 cases and 116 controls) were participating in the study. Prior two or more induced abortions [AOR = 3.95:95% CI: 1.22–13.05], previous history of caesarean section [AOR = 3.4:95% CI: 1.11–10.94], marital status (being single) [AOR = 4.04:95%CI: 1.23–13.21], reporting prior recurrent sexual transmitted infection [AOR = 2.25:95%CI: 1.00–5.51], prior history of tubal surgery [AOR = 3.32:95%CI: 1.09–10.13], were more likely to have an ectopic pregnancy with their respective AOR with 95%CI.

**Conclusion:**

It was found that having a history of more than two induced abortions during previous pregnancies, marital status (single), recurrent sexual transmitted infection, prior history of tubal surgery and experiencing prior caesarean section were found to be determinants of ectopic pregnancy. Hospitals should give emphasis on prevention and early detection of risks of ectopic pregnancy and create awareness in order to reduce the burden of ectopic pregnancy.

**Supplementary Information:**

The online version contains supplementary material available at 10.1186/s12884-021-03618-7.

## Background

Ectopic pregnancy (EP) is an abnormal condition in which implantation of the blastocyst occurs outside the endometrium of the uterus. These abnormal sites of implantation in decreasing order of frequency include uterine tube (tubal pregnancy), abdominal cavity or on the mesentery (abdominal pregnancy), and in the ovaries (ovarian pregnancy) [1, 2]. Blastocysts that do not implant in the uterine wall are generally unable to develop normally because; the space is incapable for developing blastocyst. Ectopic pregnancy can cause ruptures of fallopian tube, cervix and abdomen on which they are implanted. Rupture of ectopic pregnancy result in severe bleeding, many organ damage, and maternal mortality [3–5]. It is obstetrical and gynecological important, especially in the developing world, because of the high maternal morbidity and mortality associated with it and the enormous threat to life particularly in the first trimester pregnancy [2]. It occurs in approximately 1–2% of pregnancies [4–7]. It is one of the top leading causes of maternal mortality in the first trimester and accounts for 10–15% of all maternal deaths [7]. Ectopic pregnancy is the leading cause of maternal morbidity and mortality worldwide [8].

In western world, the prevalence of ectopic pregnancy is approximately 2% in the general population, but as high as 20% in patients who have undergo tubal surgery, previous ectopic pregnancy [8]. The prevalence of ectopic pregnancy has an increasing trend during the last three decades throughout the world especially in developing countries where early diagnosis is low [3].

The causes of ectopic pregnancy are not well understood. However, multiple risk factors have been associated with ectopic pregnancy, although some patients may not have any risk factor to developed ectopic pregnancy. The main function of the oviduct is to provide the optimal environment for the transport and maturation of ovum and sperm for the establishment of pregnancy. Most data suggest that ectopic pregnancy causes from both abnormal zygote transport and change in the tubal environment, which enables abnormal implantation to occur [9–11].

In spite of different research done on the prevalence of ectopic pregnancy, however, the determinants of ectopic pregnancy are not well understood and few researches published in our country were based on secondary data covering small scale area and the study area has different characteristics cultural, religious, socio-demographic characteristics, sexual behavior, beliefs, contraception usage and practice from other area. The study was aimed to identify risk factors of ectopic pregnancy among pregnant women attending referral hospitals in Southwestern part of Oromia Regional state, Southwest Ethiopia.

This study result would worth to detect the potential risk factors of ectopic pregnancy in the study setup which would have further advantages to minimize morbidity and mortality of patients due to ectopic pregnancy. With regard to the preventable factors associated with ectopic pregnancy in the current population, this study is an important piece of work that could serve as an important source of information to design prevention strategies or to conduct further investigations.

## Methods

### Study setting, design and population

A multi-centered hospital-based case control study was conducted among pregnant women attending referral hospitals in Southwestern parts of Oromia regional state, Southwest Ethiopia from June 1 to September 30, 2019. All hospitals are teaching and referral hospital that gave general and specialized clinical services including ANC, family planning, delivery service & treatment obstetric complications are some of the services provided in gynecologic and obstetric ward. These services have been delivered by senior midwives, gynecologists/obstetricians. All pregnant women attending gynecology and obstetrics department of JMC (Jimma Medical Center), WURH (Wollega University Referral Hospital), NRH (Nekemte Referral Hospital), AURH (Ambo Referral Hospital) and MKRH (Mettu Karl Referral Hospital) during the four-month study period were source population.

Study population: For cases all pregnant women who had been confirmed by ultrasound and HCG to have EP in the inpatient department of gynecology and obstetrics of each hospital were recruited. For controls: Controls were sampled pregnant women confirmed by ultrasound and HCG to have intra uterine pregnancy at the prenatal clinic in department of gynecology and obstetrics of each hospital.

### Eligibility criteria

#### Inclusion criteria for cases

admitted women who had been confirmed by ultrasound and HCG to have EP in the inpatient department of gynecology and obstetrics of each hospital. For controls: Controls were sampled pregnant women confirmed by ultrasound and HCG to have intra uterine pregnancy at the prenatal clinic in department of gynecology and obstetrics of each hospital.

#### Exclusion criteria for both cases and controls

Women with serious medical conditions and couldn’t give consent were excluded from the study.

#### Case definition

Case pregnant women diagnosed by hCG and ultrasound to have ectopic pregnancy confirmed by Obstetrician/gynecologist [12].

#### Control

Pregnant women diagnosed by hCG and ultrasound to have intrauterine pregnancy confirmed by Obstetrician/gynecologist [12].

### Sample size and sampling procedure

The required sample size was determined by using Epi-info version 7 statistical software for unmatched case-control study design. Results from similar studies were used to approximate the sample size in different potential risk factors of ectopic pregnancy. In a study report from India prior tubal surgery was a significant risk factor for ectopic pregnancy [13]. A case control study in western Ethiopia at Nekemte hospital marital status was a significant risk factor for ectopic pregnancy [14]. Similarly, case control study done in Turkey Ankara previous history of ectopic pregnancy was a significant risk factor for ectopic pregnancy [15]. Using these reports as starting point, similar assumptions P1: proportion among cases and p**2:** proportion of among controls AOR: Adjusted odds ratio at 95% (**Zα/2** = 1.96) level of confidence, Power of study = 80% Ratio of cases to controls = 1:2 (Table 1).

**Table 1:** Epi Info sample size calculation

Exposure variables	Proportion ion among cases	Proportion on among controls	AOR	Sample size		Final Sample Adding 10% nonresponse
				Cases	Controls	
Pervious History of ectopic pregnancy	9.7	1.3	13.1	53	106	177
Single marital status	25	3	10.8	32	64	105
Pervious tubal surgery	44	3	14	25	50	84

From the above three significant risk factors of ectopic pregnancy, previous history of ectopic pregnancy gives the large sample size which gives total of 177 study participants (59 cases and 118 controls). In the selected five referral hospitals the number of pregnant. Women registered during the 2018 G. C HMIS report over 4 months at JMC, WURH, NRH, AURH and MKRH were 1707, 1085, 679, 1489 and 1219 respectively.

The calculated sample size was proportionally allocated based on the estimated number of pregnant women in selected referral hospitals. Therefore (16 cases and 32 controls) from JMC, (10 cases and 20 controls) from WURH, (7 cases and 14 controls) from NRH, (14 cases and 28 controls) from AURH and (12 cases and 28 controls) from MKRH. Then, the study participant was selected using consecutive sampling technique.

### Data collection tools and procedures

The data were collected by face to face interview using semi structured questionnaire addressing socio-demographic and obstetric, gynecologic, behavioral, surgical history and contraceptive characteristics of study participants which was developed after reviewing different literatures. Fifteen trained data collectors and five supervisors were involved in the process.

### Data quality control

The urine sample collection was done through standardized, and sterile technique by professional laboratory technologists, ultrasound was calibrated before the procedure. The diagnosis of pregnancy was confirmed by Trans abdominal ultrasonography combined to the hCG.

Data quality was ensured during data collection, coding, entry and analysis. During data collection adequate training and follow up was provided to data collectors and supervisors. Incomplete checklists were returned back to the data collector for completion. Codes were given to the questionnaires and during the data collection so that any identified errors was traced back using the codes.

### Data processing and analysis

Collected data were rechecked for completeness, consistency and coded before data entry. Data were entered using Epi data version 3.1 and data from five hospitals were merged together, and then exported to the Statistical Package for Social Science (SPSS) version 23 for analysis. Descriptive analysis was conducted to explore the data and present some variables. Bi-variable binary logistic regression analysis was executed to select candidate variable for multivariable binary logistic regression to identify the predictors. Variables with *p*-value of less than 0.25 were selected for multivariable logistic regression. Odds ratio (OR) and 95% confidence intervals (CI) were used to describe the association between ectopic pregnancy and potential risk factors. Variables with a *p*-value < 0.05 in multi-variable analysis was considered as a significant risk factor for ectopic pregnancy.

## Results

### Socio-demographic characteristics

In this prospective case control study conducted over four-months from June 1 to September 30, 2019 at five government referral hospitals found in Southwestern part Oromia, Ethiopia. A total of 174 pregnant women; 58 Cases (EP) and 116 Controls (IUP) were participated. The mean age was 26 (± 5.54), 26 (± 4.87 years for cases and controls respectively.

Almost two-third (63.8%) of cases and 79 (68.1%) of controls were aged between 21 and 30 years. Eighteen (31%) cases and 39(33%) of controls were orthodox in religion and 37(63.8%) of cases and 79 (68.1%) of controls were Oromo by their ethnicity and 37 (63.8%) cases and 95(81.9%) of controls were married. About 18 (31%) cases and 46 (39.7%) controls were house wives in occupation (Table 2).

**Table 2:** Socio-demographic characteristics of participants at referral hospitals in Southwestern part of Oromia region, Southwest Ethiopia (*n* = 174), September 2019

Characteristics	Category	Cases (*N* = 58) N (%)	Controls (*N* = 116) N (%)
Age in year	<=20	10 (17.2%)	14 (12.1%)
	21–30	37 (63.8%)	79 (68.1%)
	> = 30	11 (18.9%)	23 (19.8)
Residence	Urban	27 (46.6%)	54 (46.6%)
	Rural	31 (53.4%)	62 (53.4%)
Religion	Orthodox	18 (31.0%)	39 (33.6%)
	Muslim	15 (25.8%)	29 (25.0%)
	Protestant	20 (34.4%)	33 (28.4%)
	Others	5 (8.6%)	15 (12.9%)
Ethnicity	Oromo	37 (63.8%)	79 (68.1%)
	Amhara	11 (18.9%)	24 (20.7%)
	Dawuro	5 (8.6%)	7 (6.0%)
	Gurage	5 (8.6%)	6 (5.2%)
Marital status of	Single	9 (15.5%)	7 (6.0%)
Respondent	Married	37 (63.8%)	95 (81.9%)
	Others	12 (13.7%)	14 (12.08%)
Educational status	Can’t read &write	6 (10.3%)	5 (4.3%)
	Read &write only	10 (17.2%)	17 (14.7%)
	Grade 1–8	18 (31.0%)	48 (41.4%)
	Grade 9–12	15 (25.8%)	32 (27.3%)
	Diploma and above	9 (15.5%)	14 (12.1%)
Occupational	Housewife	15 (25.8%)	38 (32.7%)
status of	Farmer	13 (22.1%)	22 (18.9%)
Respondent	Gov’t employee	13 (22.4%)	25 (21.6%)
	NGO	4 (6.8%)	10 (8.6%)
	Merchant	6 (10.3%)	10 (8.6%)
	Laborer	7 (12.1%)	11 (9.5%)
Income in ETB	< 1000	14 (24.1%)	23 (20.6%)
	1001–2000	10 (20.7%)	23 (19.8%)
	2001–3000	15 (25.7%)	26 (22.4%)
	3001–4000	12(10.3%)	25(20.6%)
	> 4001	7 (12.1%)	19 (16.4%)

### Behavioral characteristics

Only one case (1.7%) and two controls (1.7%) had occasional history of cigarette smoking and only 18(31.1%) cases and 34(29.3%) controls history of occasionally alcohol consumption before current pregnancy (Table 3).

**Table 3:** Behavioral characteristics of participants in Southwestern referral hospitals in Oromia regional state, Southwest Ethiopia (*n* = 174), September 2019

Habits	Category	Cases (*N* = 58), N (%)	Controls (*N* = 116, N (%)
History of cigarette	Non smoker	57 (98.2%)	114 (98.3%)
	Occasional smoker	1 (1.7%)	2 (1.7%)
History of alcohol d	Non drinker	40 (68.9%)	82 (70.7%)
	Occasional drinker	18 (31.1%)	34 (29.3%)

### Obstetrics and surgical history of participants

As indicated in the Table 4, three of the cases (5.1%) and another three women in the control group (2.6%) had prior history of ectopic pregnancy. Seven women in each of the study groups (12.0% of the cases and 6.0% of the controls) had more than two prior history of spontaneous abortion. Similarly, 8 (13.8%) cases and 6 (5.1%) controls reported two or more prior history of induced abortions. This study shows that 10(17.2%) of cases and 6(5.1%) controls had caesarean section before current pregnancy. Eleven (18.1%) of cases and 6(5.2%) controls had at least one tubal pregnancy before current pregnancy for any reason (Table 4).

**Table 4:** Obstetrics and surgical history of participants in Southwestern referral. Hospitals in Oromia regional state, Southwest Ethiopia (*n* = 174), September 2019

Characteristics (***N*** = 174).	Category	Cases (*N* = 58) N (%)		Controls (*N* = 116,N (%)
Prior history of ectopic	Yes	3	(5.1%)	3 (2.6%)
Pregnancy	No	55 (94.8%)		113 (97.4%)
Prior history of spontaneous	0	42 (72.4%)		97 (83.6%)
Abortion	1	9	(15.5%)	12 (10.3%)
	> = 2	7	(12.0%)	7 (6.0%)
Prior history of Induced	0	40 (68.9%)		96 (82.8%)
Abortion	1	10 (17.2%)		14 (12.1%)
	> = 2	8	(13.8%)	6 (5.1%)
Prior history of caesarean	Yes	10 (17.2%)		6 (5.1%)
Section	No	48 (82.7%)		110 (94.8%)
Prior history of	Yes	1	(1.7%)	4 (3.4%)
Appendectomy	No	57 (98.3%)		112 (96.6%)
Prior history of tubal surgery	Yes	11 (18.9%)		6 (5.2%)
	No	47 (81.1%)		110 (94.8%)
Prior history of tubal ligation	Yes	5	(8.6%)	5 (4.3%)
	No	53 (91.4%)		111 (95.7%)
Parity	0	19 (32.7%)		43 (37.1%)
	1	22 (37.9%)		48 (41.3%)
	> = 2	17 (29.3%)		25 (21.6%)

### Gynecologic and contraceptive history of participants

About 36.2% (21/58) of the cases and 16 (13.7%) controls had prior history of a recurrent.

STD/STI. Majority 42 (72.4%) of the cases and 92 (79.3%) controls had prior history of oral contraceptive use. Only 6 (10.3%) cases and 15(12.9%) controls had history of IUCD use.

Twenty (34.4%) cases and 18 (10.8%) controls reported practice of emergency contraceptives pills use before the current conception (Table 5).

**Table 5:** Gynecologic and contraceptive history of participants in Southwestern referral hospitals in Oromia regional state, Southwest Ethiopia (*n* = 174), September 2019

Characteristics	Category	Cases (*N* = 58),	Controls (*N* = 116)	
		N (%)	N (%)	
Prior recurrent STD/STI	Yes	21 (36.2%)	16	(13.7%)
	No	37 (63.7%)	100 (86.2%)	
Prior history condom	Yes	16 (27.5%)	28	(33.6%)
Usage	No	42 (72.4%)	88	(75.8%)
Prior history of IUCD	Yes	6 (10.3%)	15	(12.9%)
Usage	No	52(89.7%)	101 (87.1%)	
Prior history of OCP	Yes	42 (72.4%)	92	(79.3%)
	No	16 (27.6%)	24	(20.7)
Prior history of Injectable Yes		25 (43.1%)	50	(43.1%)
	No	33 (56.9%)	66	(56.9%
Prior history of implant	Yes	15 (25.9%)	34	(29.3%)
Use	No	43 (74.1%)	82	(70.7%)

### Factors associated with ectopic pregnancy

Findings from bi-variable logistic regression analysis showed that marital status, prior history of induced abortions, prior history of spontaneous abortions, prior history of tubal surgery, prior history of caesarean section, prior history of tubal ligation and history of recurrent STD/STI had associated with ectopic pregnancy with p-value of < 0.25, However, in.

multivariable regression analysis, history of two or more induced abortions [AOR = 3.42:95%CI: 1.06–11.05], prior history of caesarean section [AOR = 3.48:95% CI: 1.14–10.13], prior history of tubal surgery [AOR = 3.32:95%CI: 1.09–10.13], marital status (being single) [AOR = 3.23:95%CI:1.02–10.22], prior recurrent STD/STI [AOR = 3.08:95%CI: 1.38–6.88} remained statistically significant risk factor for ectopic pregnancy (Table 6).

**Table 6:** Bivariate and multivariate logistic regression

Variables	Category	Status of ectopic pregnancy		COR (95% C.I)	AOR (95% C.I) *P* value
		Cases (*N* = 58) N (%)	Controls (*N* = 116) N (%)		
Marital status of respondent	Single	9 (15.5%)	7 (6.0%)	3.31[1.14–9.51]	**3.22**[1.02:10.22] 0.04*
	Married	37(63.8%)	95(81.9%)	1	
	Others	12(13.7%)	14(12.08%)	2.2[0.93–5.1]	2.0[0.75–5.36] 0.16
Prior history of spontaneous abortions	0	42(72.4%)	97 (83.6%)	1	
	1	9 (15.5%)	12(10.3%)	1.73[0.67–4.42]	1.48[0.50–4.37] 0.43
	> = 2	7(12.0%)	6(12.0)	2.3[0.76–6.97]	1.68[0.45–0.62] 0.48
Prior history of induced abortion	0	40(68.9%)	96 (82.8%)	1	
	1	10(17.2%)	14 (12.1%)	1.71[0.71–4.18]	1.72[0.66–4.51] 0.26
	> = 2	8 (13.8%)	6 (5.1%)	3.2[1.04–9.81]	**3.42**[1.06–11.1] 0.04*
Previous history of caesarean section	Yes	10(17.2%)	6 (5.1%)	3.82[1.49–12.2]	**3.48**[1.14–10.6] 0.028*
	No	48(82.7%)	110 (94.8%)	1	
Prior history of tubal surgery	Yes	11(18.9%)	6 (5.2%)	4.3[1.49–12.28]	**3.32**[1.1–10.13] 0.035*
	No	47(81.1%)	110(94.8%)	1	
Tubal ligation	Yes	5 (8.6%)	5 (4.3%)	2.64[0.64–10.3]	1.32[0.26–6.64) 0.73
	No	53(91.4%)	111 (95.7%)	1	
Prior STD/STI	Yes	21(36.2%)	16 (13.7%)	3.55[1.67–7.52]	**2.35**[1.0–5.51] 0.049*
	No	37(63.7%)	100 (86.2%)	1	

*value statistically significant, *AOR* adjusted odds ratio, *COR* crude odds ratio, *CI* confidence interval, 1-reference

## Discussion

This was a multi-centered hospital based case control study which, was aimed to identify determinants of ectopic pregnancy among pregnant women attending referral hospitals in Southwestern parts of Oromia regional state, Southwest Ethiopia. Being single were independent predictors of ectopic pregnancy. A similar association was reported in studies done in west Ethiopia Nekemte and Uganda [14, 16]. The association between being single and ectopic pregnancy infection could be explained by the fact that single women engaged in multiple sexual partners following successive infection, ascending infection result in adhesions, impede the morula retention of movement causing implantation in the tube and other site.

Having more than two times history of induced abortion found was statistically significant relation with ectopic pregnancy. This finding was supported by a study done in; India, Tigray, Ethiopia and Nigeria [17–19]. The association might be explained by most abortions are illegal different countries and usually performed in poor aseptic conditions. Thus, increasing post-abortion sepsis risk and subsequent PID.

Women who had a prior history of recurrent STI were significantly associated with ectopic pregnancy. This finding was similar to studies done in Ethiopia, Ghana [20]. The association between STD/STI and ectopic pregnancy might be successive infection, ascending infection result in salphingitis leads to tubal dysfunction, decrease cilia density; ciliary beat this result in retention of morula in the fallopian tube and implantation of blastocyst in the fallopian tube and other site.

Women having at least one caesarean section for previous pregnancy were independently associated with ectopic pregnancy. This study supported by a study done in Turkey [15]. The underlying mechanism of association between previous caesarean section and occurrence of ectopic pregnancy is might be due to increased pelvic infection and adhesion after caesarean section which disturbs the micro environment of the tube and implantation of blastocyst in the tube.

In the present study, women who had a prior history of tubal surgery were statistically significant with ectopic pregnancy. This study is supported by study done in Egypt and Uganda [16]. The association might be explained by the scar on the Fallopian tube may interfere with the ovum transport and implantation of blastocyst in the Fallopian tube.

I did not find any association between appendectomy, prior use of IUCD, cigarette smoking, alcohol drinking and previous history of ectopic pregnancy, previous tubal surgery with present study, probably the number of the studied participants was too small.

### Limitations and strengths of the study

Due to a small number of cases obtained from each hospital, this study did not compare among the five hospitals with regard to risk factors of EP and study assesses history of exposure retrospectively, it may be prone to recall and selection bias by nature during the data collection time. The study has some strengths this study used the primary data from the participants. Further, the study was multi-centered hospital based case control.

## Conclusions

It was found that having a history of more than two induced abortions during previous pregnancy, marital status (single), experiencing at least one caesarean section for previous pregnancies, prior history of STD/STI and using emergency contraceptive pills during the cycle of conception were found important determinants of ectopic pregnancy in the study population. Women with history of previous induced abortion and previous caesarean section STD/STI should be followed up carefully, even in the absence of symptoms should always be counseled about the possibility of ectopic pregnancy and the associated risks.

## Supplementary Information

**Additional file 1.**

## Data Availability

The datasets used and/or analyzed during the current study are available from the corresponding author on reasonable request.

## Abbreviations

AOR: Adjusted odd ratio
AURH: Ambo University Referral Hospital
COR: Crude odd ratio
EP: Ectopic pregnancy
HCG: Human chorionic gonadotropin
HMIS: Health Management Information system
IUP: Intra uterine pregnancy
JMC: Jimma Medical Center
MKRH: Mettu Karl Referral Hospital
NRH: Nekemte Referral Hospital
STD: Sexual transmitted diseases
STI: Sexual transmitted infection
