# Supplementary material for: Determinants of ectopic pregnancy among pregnant women attending referral hospitals in southwestern part of Oromia regional state, Southwest Ethiopia: a multi-center case control study
Source: BMC Pregnancy Childbirth. 2021 Feb 12;21:130. doi: 10.1186/s12884-021-03618-7 (PMC7881641; doi:10.1186/s12884-021-03618-7)
Supplement: Supplementary file 1 — Additional file 1. [file 12884_2021_3618_MOESM1_ESM.docx]

**Annex II. English Versions Questionnaire**

**Part I Socio-demographic characteristics of the respondents.**

| Code | Variable | Response | Remark/note |
| --- | --- | --- | --- |
| 101 | State of pregnancy | 1. IUP 2. Ectopic pregnancy |  |
| 102 | If **Qno 1** is Ectopic pregnancy where is the site of pregnancy? | 1. Tubal 2. Abdominal 3. Ovarian 4. Cervix 5. Other specify--------------- |  |
| 103 | If **Qno2** is tubal where is the site | 1. ampulla  2. isthmus  3. fimbrie  4. corneal |  |
| 104 | Age | ---------------in years |  |
| 105 | Residence | 1. Urban  2. Rural |  |
| 106 | Marital status | 1. Single 2. Married 3. Widowed 4. Divorced |  |
| 107 | Educational level | 1. Illiterate 2. Read and Write 3. 1-8t^h^ grade 4. 9-12^th^ grade 5. College or University |  |
| 108 | Occupation | 1. Housewife 2. Farmer 3. Government employee 4. Non- government employee 5. Merchant 6. Daily laborer |  |
| 109 | Ethnicity | 1. Oromo  2. Amhara  3. Dawuro  4. Guraghe  5. Other/ specify------------ |  |
| 110 | Religion | 1. Orthodox  2. Muslim  3. protestant  4. Other/ specify |  |
| 111 | Monthly average income in ETB | ------------------- |  |

**Part II Past Obstetrics and surgical History**

| Code | Variable | Response | Skip |
| --- | --- | --- | --- |
| 112 | Do you have Pervious history of Ectopic pregnancy? | 1. No 2. Yes |  |
| 113 | Pervious history of spontaneous abortion? | 1. 0  2. 1  3. 2 and above |  |
| 114 | Previous history of induced abortion | 1. 0  1. 1  2. 2 and above |  |
| 115 | Previous history of appendectomy | 0. No  1. Yes |  |
| 116 | Previous history of tubal surgery | 0. No  1. Yes |  |
| 117 | Previous history of tubal ligation | 0. No  1. Yes |  |
| 118 | Previous history of caesarean section | 0. No  1. Yes |  |
| 119 | Parity | 1. 0  1. 1  2. 2 and above |  |
| 120 | Pervious history of recurrent STI/STD | 0. No  1. Yes |  |
| 121 | History of infertility | 0. No  1. Yes |  |
| 122 | History of condom use | 0. No  1. Yes |  |
| 123 | History of IUCD use | 0. No  1. Yes |  |
| 124 | History of OCP use | 0. No  1. Yes |  |
| 125 | History of injectable contraceptive use | 0. No  1. Yes |  |
| 126 | History of implant(implant + Jeddelle) | 0. No  1. Yes |  |

**Part IV Behavioral History**

| 127 | Cigarettes smoking | 1. non smoker  2. occasional smokers  3. regular smokers | |  |
| --- | --- | --- | --- | --- |
| 128 | If Yes to **124** how long ago did you start smoking? | ----------------years | |  |
| 129 | If yes to **124**, how often were you smoking? | 1. Daily 2. Once a week 3. 3 times per week 4. Once a month | |  |
| 130 | How many cigarettes do you smoke each day/week on average? | ----------numbers of cigarette | |  |
|  | **Alcohol status** | | | |
| 130 | Do you have a History of alcohol drinking? | | 1.Yes  2. No |  |
| 131 | If Yes to Q128 How often do you have a drink containing alcohol? | | 1 Never 2. Less than monthly 3. 2-4 times a month 4. 2-3 times a week 5. 4/more times a week |  |
|  | **Alcohol status** | | | |
| 133 | History of alcohol drink containing alcohol? | | 1 Never 2. Less than monthly 3. 2-4 times a month 4. 2-3 times a week 5. 4/more times a week |  |

**Part V HCG and ultrasound measurement**

1. HCG--------------------------------------------(mIU/mL) milli-international units per milliliter (positive or Negative)
2. Result of Ultrasound
   1. Tubal ectopic pregnancy
   2. Abdominal
   3. Ovarian pregnancy
   4. Cervical pregnancy
   5. Intrauterine pregnancy
   6. Heterotrophic pregnancy
